# Geographic variation and genetic structure in the Bahama Oriole (*Icterus northropi*), a critically endangered synanthropic species

**DOI:** 10.7717/peerj.1421

**Published:** 2015-11-26

**Authors:** Melissa R. Price, Carl Person, William K. Hayes

**Affiliations:** 1 Department of Natural Resources and Environmental Management, University of Hawaiʻi at Mānoa, Honolulu, HI, United States; 2 Kewalo Marine Laboratory, Pacific Biosciences Research Center, University of Hawaii at Manoa, Honolulu, HI, United States; 3 Department of Earth and Biological Sciences, Loma Linda University, Loma Linda, CA, United States

**Keywords:** Population genetics, Conservation genetics, Ornithology, Single-island endemic species, Non-migratory subtropical species

## Abstract

Bird species may exhibit unexpected population structuring over small distances, with gene flow restricted by geographic features such as water or mountains. The Bahama Oriole (*Icterus northropi*) is a critically endangered, synanthropic island endemic with a declining population of fewer than 300 individuals. It now remains only on Andros Island (The Bahamas), which is riddled with waterways that past studies assumed did not hinder gene flow. We examined 1,858 base pairs of mitochondrial DNA sequenced from four gene regions in 14 birds (roughly 5% of the remaining population) found on the largest land masses of Andros Island (North Andros and Mangrove Cay/South Andros). We sought to discern genetic structuring between the remaining subpopulations and its relationship to current conservation concerns. Four unique haplotypes were identified, with only one shared between the two subpopulations. Nucleotide and haplotype diversity were higher for the North Andros subpopulation than for the Mangrove Cay/South Andros subpopulation. Analysis of molecular variance (AMOVA) yielded a Wright’s fixation index (*F*_st_) of 0.60 (*P*_Fst_ = 0.016), with 40.2% of the molecular variation explained by within-population differences and 59.8% by among-population differences. Based on the mitochondrial regions examined in this study, we suggest the extant subpopulations of Bahama Oriole exhibit significant population structuring over short distances, consistent with some other non-migratory tropical songbird species.

## Introduction

Bird species may exhibit unexpected population structuring, with gene flow limited by geographic features in ways that are sometimes unanticipated, given birds’ flight capabilities. In some cases, genetic structuring may result from ecological associations with forest or aquatic environments that lead to population structuring over small distances (Valderrama et al., 2014). For non-migratory insular bird species, the ocean appears to limit gene flow among islands (Arnaux et al., 2014; Saitoh et al., 2015) and between islands and continents (Cortes-Rodriguez et al., 2008). Constraints on gene flow may be particularly pronounced in non-migratory Neotropical bird species compared with their migratory North American relatives. Indeed, Neotropical bird species generally demonstrate intraspecific divergence that is an order of magnitude higher than North American species (2.13% vs. 0.23%; Milá et al., 2012).

The Bahama Oriole (*Icterus northropi*), one of but a very few critically endangered synanthropic bird species (Price, Lee & Hayes, 2011), is a year-round resident on Andros Island (The Bahamas), a land mass riddled with waterways that effectively divide it into multiple islands. The largest three sections, and the only ones with significant human development, include North Andros, Mangrove Cay, and South Andros. These islands are separated by channels up to 5 km wide, which in previous assessments of oriole populations were assumed not to limit dispersal (Baltz, 1997). Approximately 20 km, including two 5-km-wide channels and multiple cays, separate the southern tip of North Andros from the northern tip of Mangrove Cay. In contrast, Mangrove Cay and South Andros are separated by a mere 1–3-km-wide channel with multiple smaller cays in-between, and in this study orioles on these southern islands were deemed to be a continuous subpopulation. Considering the exceptional flight capabilities of migratory oriole species (Jaramillo & Burke, 1999), movement of orioles among all three islands seems likely.

Recently elevated to species status (American Ornithologists’ Union, 2010), *I. northropi* has a declining population of potentially fewer than 300 individuals (Price, Lee & Hayes, 2011). Historically known from only two major islands in the Bahamas, the species became extirpated from Abaco in the 1980s (Baltz, 1997; White, 1998). Two potential threats jeopardize the last remaining population on Andros: the accidental introduction of lethal yellowing, a phytoplasma which has devastated the favored breeding habitat (introduced Coconut Palms, *Cocos nucifera*) on North Andros (Price, Lee & Hayes, 2011); and the recent natural arrival of the Shiny Cowbird (*Molothrus bonariensis*), a brood parasite that targets *Icterus* (Baltz, 1995; Baltz, 1996; Wiley, 1985). In addition to these threats, coppice habitat (native dry broadleaf forest), which appears to be crucial to year-round survival of the oriole (Currie et al., 2005; Price, Lee & Hayes, 2011), continues to be destroyed for agricultural and other anthropocentric purposes (Wunderle & Waide, 1993; Thurston, 2010).

We assessed genetic variation in the Bahama Oriole for two purposes: (1) to discern genetic structuring of the two potential subpopulations (North Andros and South Andros/Mangrove Cay); and (2) to relate our findings to current conservation concerns. One useful application, for example, would be to inform planning for possible translocation of the Bahama Oriole to Abaco, where it formerly occurred.

## Methods

### Sample collection

**Figure 1:**
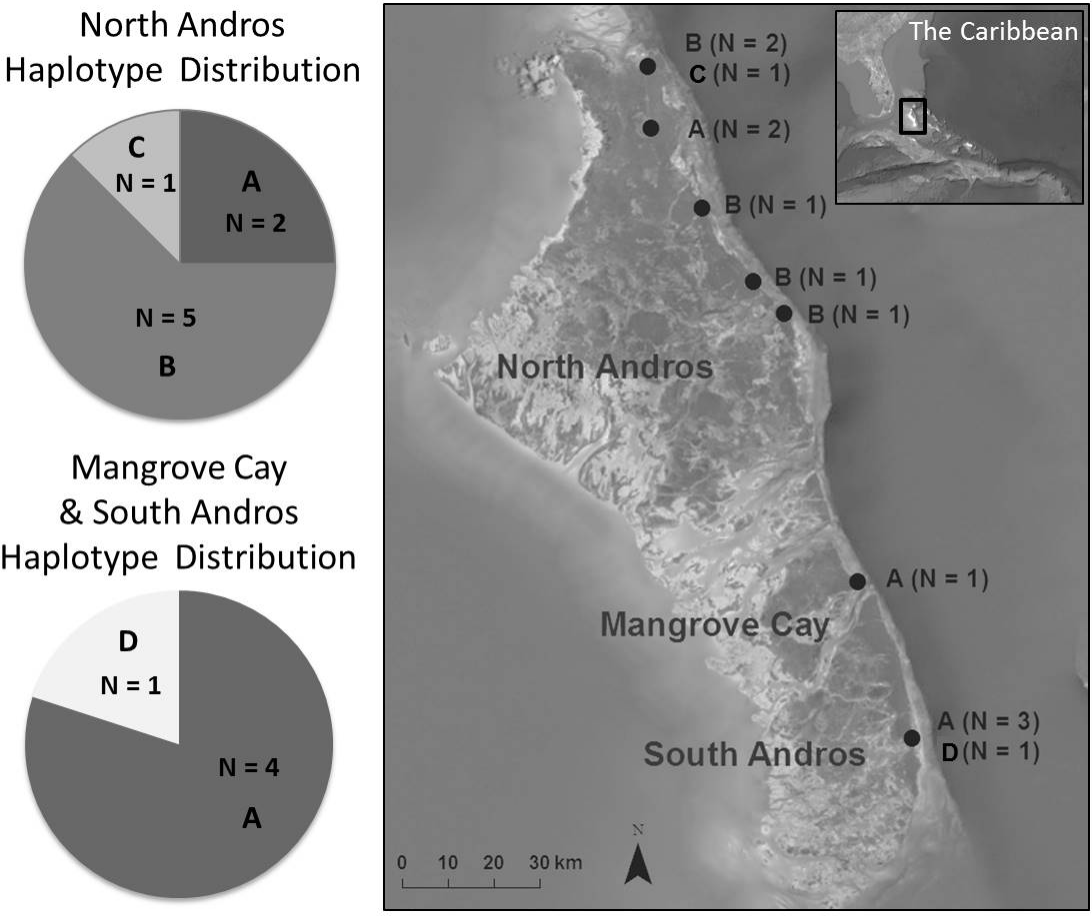
Bahama Oriole haplotype distribution among sampled localities on Andros, The Bahamas.

We collected blood and feather samples from 16 live birds captured at 11 locations throughout the Bahama Oriole’s distribution on North Andros (*N* = 11), Mangrove Cay (*N* = 1), and South Andros (*N* = 4) during the years 2009–2010 (Table 1). They were collected in accordance with all required permits (Bahamas Ministry of the Environment Permit to Conduct Scientific Research in the Bahamas; USDA/APHIS/VS Permit 108969 to import tissue samples; and IACUC protocol #8120010 approved by the Loma Linda University Institutional Animal Care and Use Committee.) This sample size, although small, represents approximately 5% of the estimated population. Samples were evenly distributed throughout the known range, where only a few pairs of orioles nest in each township or agricultural area (Fig. 1; Price, Lee & Hayes, 2011). Birds were captured by mist net using song playback (*N* = 12), or as nestlings briefly removed from two nests on North Andros (*N* = 4; see data treatment below). Captured birds were measured, sampled, banded using standard issue aluminum USGS identification bands, and immediately released. Two tail feathers were pulled from adult birds. For nestlings and adults, blood samples were obtained by pricking the brachial vein (Arctander, 1988) and collecting pooling blood with a capillary tube. Blood was immediately mixed with lysis buffer (100 mM Tris pH 8.0, 100 mM EDTA, 10 mM NaCl, 0.5% SDS; Longmire et al., 1988), and placed on ice. After transportation to the laboratory, samples were stored at −20 °C. Blood volumes collected from each individual (0.1–0.2 mL) were well below the recommended limit of <1% of the body weight for a 30–35-g bird (Gaunt & Oring, 1997). Individuals were tracked visually after sampling for a minimum of four days, and no casualties were observed.

**Table 1:** *Icterus northropi* samples used for genetic analysis and their haplotypes. Since two pairs of samples were from siblings pulled from two nests, and individuals from the same nest were subsequently found to share the same haplotypes, one sibling from each pair was excluded from further analysis. An additional individual was excluded for some analyses due to our inability to sequence ATP 6/8.

Island	Year	Haplotype	Band number
North Andros	2009	B	120239501
			120239502
		B	120239503
		B	120239504
		B	120239505
		A	120239506
		A	120239507
			120239509
		B	120239510
			120239511
		C	120239512
Mangrove Cay	2010	A	120239517
South Andros	2010	A	120239524
		D	120239526
		A	120239527
		A	120239528

### DNA isolation

DNA extractions were based on the protocol of Fetzner & Crandall (2003) with minor modifications. We diluted 2 µL of blood in 300 µL of cell lysis buffer, and then added 1.5 µL of RNAase A. Samples were placed in a 37 °C water bath for 15 min. After returning to room temperature, 100 µ L ammonium acetate was added, and samples were vortexed and centrifuged. The supernatant was poured into new tubes with 500 µL isopropanol, washed several times with 300 µL ice cold ethanol, then air dried to remove alcohol. Finally, we reconstituted samples with 30 µL TE buffer (10 mM Tris–HCl, pH 8.0, 1 mM EDTA). Similar protocols were followed for feather DNA extractions, with a few additional initial steps. Feather shafts were minced and added to 500 µL cell lysis buffer and 5 µL protinase K, then placed in a 55 °C water bath for 24 h. Following protein digestion, the above protocols for DNA extraction were followed.

We amplified mitochondrial DNA (mtDNA) sequences from the genomic DNA samples using standard polymerase chain reaction (PCR) methods, as detailed below. Four mtDNA gene regions were amplified: ATP synthase subunits 6 and 8 (ATP6/8); cytochrome *b* (cyt*b*); and NADH dehydrogenase subunit 2 (ND2), using the primers in Table 2. We chose these genes because they evolve at different but relatively high rates, they are as much as 6,000 bp away from each other, and because the use of multiple primers allowed us to compare genetic variation in the Bahama Oriole with other species (Omland, Lanyon & Fritz, 1999). Reactions of 50 µL were prepared according to manufacturer’s instructions by mixing 25 µL Maxima Hot Start PCR Master Mix (Thermo Scientific, Waltham, Massachusetts, USA), 2 µL whole genomic reconstituted DNA, 1 µL of each primer, and 21 µL nuclease-free water. PCR conditions were as follows: initial denaturation at 95 °C for 4 min, followed by 60 cycles of denaturation at 95 °C for 30 s, annealing at 50 °C for 30 s (same for all primers), and extension at 72 °C for 1 min, with a final extension at 72 °C for 15 min. PCR products were separated in non-denaturing 1.5% agarose gels, then stained with 0.05% ethidium bromide (EtBr), and visualized using an UV imager. After confirmation of the presence of DNA, PCR products were sequenced by Macrogen (Rockville, Maryland, USA).

**Table 2:** Mitochondrial DNA PCR primers used to assess geneticvariation in *Icterus northropi*.

mtDNA gene^a^	Primer sequence	Reference	bp
ATP6/8	5′AAAGCRTYRGCCTTTTAAGC	L8331	860
	5′GTTAGTGGTCAKGGGCTTGGR	CO 3.2 H936	
		(Perdices & Doadrio, 2001)	
Cyt*b*	5′TCAAACATCTCAACCTGATGA	703Bot	667
	5′GGCAAATAGGAAGTATCATTC	MV16	
		(Pook, Wuster & Thorpe, 2000)	
ND2	5′TATCGGGCCCATACCCCGAAA	L5215	331
	5′CCTTGAAGCACTTCTGGGAAT	H57761	
		(Hackett, 1996)	

**Notes.**

a ATP6/8, ATP synthase, subunits 6 and 8; Cyt*b*, cytochrome *b*; ND2 NADH dehydrogenase, subunit 2.

### Population genetics analysis

To examine possible genetic structure, we compared two arbitrary subpopulations: North Andros and South Andros/Mangrove Cay. We pooled the South Andros and Mangrove Cay populations for analysis because of the small number of samples available, and because stepping stone cays with suitable foraging habitat are much closer together between Mangrove Cay and South Andros than between North Andros and Mangrove Cay. Multiple alignments for each gene region were performed in CLUSTALX version 2.1 (Larkin et al., 2007). Sequences were edited by visualizing sequence trace files and using text editing software. Haplotypes were determined using DnaSP 5.10 (Librado & Rozas, 2009). Since two pairs of samples were from siblings pulled from two nests on North Andros, and were subsequently found to share the same haplotypes, one sibling from each pair was excluded from further analysis. We were also unable to sequence ATP 6/8 for an additional individual from North Andros, and therefore excluded it from some analyses. To quantify levels of genetic variation, haplotype variability was calculated as the number of haplotypes (*N*), haplotype diversity (*h*), and nucleotide diversity (*π*; Nei, 1987, Eq. 8.4 and 10.6, respectively) using ARLEQUIN 3.5 (Excoffier & Lischer, 2010). We explored relationships among individuals by constructing haplotype networks using the method of statistical parsimony implemented in TCS 1.21 (Clement, Posada & Crandall, 2000). This network method (Templeton, Crandall & Sing, 1992) allows for the non-bifurcating genealogical relationships often found in mitochondrial DNA at population-level studies (Crandall & Templeton, 1996). Analysis of molecular variance (AMOVA; Excoffier, Smouse & Quattro, 1992), performed with ARLEQUIN, was used to estimate *F*_st_ statistics, which synthesize information on nucleotide differences among haplotypes both within and among populations. Wright’s fixation index (*F*_st_) ranges from 0 to 1, with values less than 0.01 indicating little divergence among populations, and values above 0.1 indicating substantial divergence among populations.

## Results

### Patterns of sequence variation

Using a BLAST search, we found that sequences obtained in this study were consistent with previously published sequences for *Icterus northropi* or close relatives (Genbank accession numbers, respectively: ATPase 6/8, 95% identity with 97% of the query cover for *I. dominicensis*, AF109419.1, Lovette, Bermingham & Ricklefs, 1999; cytochrome *b*, 99–100% identity with 100% query cover for *I. northropi*, AF099287, Omland, Lanyon & Fritz, 1999; NADH2, 98% identity with 100% query cover for *I. dominicensis*, AF099325, Omland, Lanyon & Fritz, 1999). This indicates the DNA fragments amplified in the present study (GenBank accession numbers: ATPase 6/8: JN020618–JN020630; cytochrome *b*: JN020603–JN020617; NADH2: JN020589–JN020630) represent the intended mitochondrial targets rather than nuclear homologues.

We amplified 162 bp from ATP6, 698 bp from ATP8, 667 bp from cyt*b*, and 331 bp from ND2, for a total of 1,858 bp. Of these, 1,852 (99.7%) characters were constant, and 6 (0.3%) characters were variable and informative. Variation within the combined four gene regions included 5 transitions and 1 transversion, consistent with the expectation that transitions outnumber transversions, and consistent with the degree of mitochondrial variation in members of the genus *Icterus* (Lovette, Bermingham & Ricklefs, 1999). Four unique haplotypes were identified among the 13 individuals in which all genes amplified. The most common haplotype (A, *N* = 6) was found in both subpopulations, and the second most common haplotype (B, *N* = 5) was found only in the northern subpopulation (Fig. 1). Two haplotypes existed in only one individual each. A total of three haplotypes occurred on North Andros, and two in the southern islands (Fig. 1). Haplotype network construction resulted in a single network for the two subpopulations.

The AMOVA for combined sequences (Table 3) yielded a Wright’s fixation index (*F*_st_) of 0.60 (*P*_Fst_ = 0.016), with 40.2% of the molecular variation explained by within-population differences and 59.8% by among-population differences. Nucleotide and haplotype diversity (Table 4) were higher for the northern subpopulation (combined sequences: *π* = 0.33; *h* = 0.61) than for the southern one (*π* = 0.067; *h* = 0.40).

**Table 3:** Analysis of molecular variance. Based on four mtDNA gene regions (ATP synthase subunits 6 and 8, cytochrome *b*, NADH dehydrogenase subunit 2) for the two extant subpopulations of Bahama Oriole (North Andros and Mangrove Cay/South Andros).

Source of variation	df	Sum of squares	Variance	% of variation	*F*_st_	*P*
Among populations						
ATP 6	1	0.05	−0.01	−6.9	−0.07	1.00
ATP 8	1	5.19	0.78	65.5	0.66	0.02
Cyt*b*	1	0.13	0.01	12.6	0.13	0.35
ND2	1	1.94	0.28	68.5	0.69	0.02
Combined sequences	1	7.09	1.04	59.8	0.60	0.02
Among individuals						
ATP 6	11	0.88	0.08	106.9		
ATP 8	11	4.50	0.41	34.5		
Cyt*b*	12	0.80	0.07	87.4		
ND2	12	1.56	0.13	31.5		
Combined sequences	11	7.68	0.70	40.2		
Total						
ATP 6	12	0.92	0.07			
ATP 8	12	15.86	1.50			
Cyt*b*	13	0.93	0.07			
ND2	13	3.50	0.41			
Combined sequences	12	14.77	1.74			

**Table 4:** Comparison of nucleotide (*π*) and haplotype (*h*) diversity estimates among four mitochondrial genes (cytochrome *b*, NADH dehydrogenase subunit 2, ATP synthase subunits 6 and 8) for two subpopulations of the Bahama Oriole (North Andros and Mangrove Cay/South Andros).

Geographic region and Gene	*N*	No. base pairs	No. haplo types	*π*	*h*
North Andros					
ATP 6	8	162	2	0.25	0.25
ATP 8	8	698	2	0.43	0.43
Cyt*b*	9	667	1	0.00	0.00
ND2	9	331	2	0.39	0.39
Combined	8	1,858	3	0.33	0.61
Mangrove Cay & South Andros					
ATP 6	5	162	1	0.00	0.00
ATP 8	5	698	1	0.00	0.00
Cyt*b*	5	667	2	0.40	0.40
ND2	5	331	1	0.00	0.00
Combined	5	1,858	2	0.07	0.40
Total population					
ATP 6	13	162	2	0.15	0.15
ATP 8	13	698	2	0.54	0.54
Cyt*b*	14	667	2	0.14	0.14
ND2	14	331	2	0.54	0.54
Combined	13	1,858	4	0.41	0.68

**Notes.**

Nucleotide (*π*) and haplotype (*h*) diversity are reported as means; *N* number of individuals.

### Comparison of gene regions

Three of four gene regions, totaling 1,191 bp, lacked any variation in the southern subpopulation, and the fourth gene region (667 bp) lacked variation in the northern subpopulation (Table 4). Cytochrome *b* had the highest nucleotide diversity of the four gene regions examined for the southern islands, and ATP8 had the highest nucleotide diversity of the North Andros gene regions.

## Discussion

Based on examining four mitochondrial genes, the extant Bahama Oriole population on Andros appears to have significant population structuring. The two subpopulations we sampled appear to be relatively diverse compared with other bird species (Sgariglia & Burns, 2003; Fleischer et al., 2007; Cortes-Rodriguez et al., 2008; Cortéz-Rodríguez et al., 2013; Cadena et al., 2011). Given the oriole’s estimated population size of fewer than 300 individuals, management interventions may be necessary to maintain this diversity in the future, as decreased mate choice and increased inbreeding may result in a loss of genetic variation and decreased fitness over time (Hedrick, 1998; Mitton, 1998; Grant, Grant & Petren, 2001; Spielman et al., 2004).

The southern subpopulation of orioles, which is considerably smaller than the northern population (Price, Lee & Hayes, 2011), had substantially lower nucleotide diversity than the northern subpopulation in the individuals we tested. Further work is needed to confirm this result, given the dire implications of this finding in small populations, which are more vulnerable to inbreeding. Low nucleotide diversity suggests fitness may decrease over time in the southern population if alleles are lost due to genetic drift (Grant, Grant & Petren, 2001; Spielman et al., 2004). Continued monitoring of the southern subpopulation especially is desirable to determine levels of inbreeding and reproductive success, particularly after the recent habitat destruction on South Andros (Thurston, 2010), which may have further exacerbated population decline.

### Population differences and gene flow

We found some evidence for genetic differences between Bahama Orioles from North Andros and those from the southern islands of Mangrove Cay and South Andros. Although our sample sizes were relatively small, we collected samples from close to 5% of the estimated species population, with samples evenly distributed from north to south, and still found significant genetic structure. Only one haplotype was shared between regions, with two individuals on North Andros sharing a haplotype otherwise found only on the southern islands. A majority of the diversity was between the two subpopulations (59.8%), with 40.2% of the genetic variation explained by differences among individuals. The calculated *F*_st_ value (0.60; *P*_Fst_ = 0.016) for the two subpopulations indicates significant genetic divergence between localities despite the relatively small sample size.

Despite the migratory capabilities of the continental members of the genus *Icterus*, such as the Orchard Oriole (*I. bullockii*) and Baltimore Oriole (*I. galbula*), the Bahama Oriole and its close relatives on the islands of Cuba, Puerto Rico, and Hispaniola do not migrate (Jaramillo & Burke, 1999). Based on the results of this study, we suggest dispersal among islands, at least in females, may also be limited in *I. northropi*. The degree of genetic differentiation over the relatively short distance between North Andros and Mangrove Cay that was found in this species is typical of other non-migratory avian species, and may be associated with gap avoidance (Hackett, 1996; Robertson & Radford, 2009). Differentiation was much smaller, however, than that of two Lesser Antilles oriole species separated by 27 km of water (Lovette, Bermingham & Ricklefs, 1999), which gives some indication of the limits to which non-migratory oriole species ordinarily disperse. Future studies of the Andros population should include both mitochondrial and nuclear regions and a larger number of samples to discern the extent to which these smaller waterways hinder dispersal of male and female orioles.

### Comparison of gene regions

Examination of multiple gene regions offers several benefits in population-level studies, primarily because it provides the greatest possible explanatory power when examining relationships (Nixon & Carpenter, 1996). Corroboration between data sets increases their significance, and disagreement can provide important insights into evolutionary processes (Miyamoto & Fitch, 1995; Wiens, 1998). The use of four mitochondrial gene regions allowed us to identify diversity and relationships that a single gene assessment would have overlooked. Three of four gene regions lacked any genetic variation in the southern subpopulation, and the remaining gene region lacked genetic variation in the North Andros subpopulation. Nucleotide diversity for the combined regions was low for the southern subpopulation, but we would have failed to identify any existing diversity without examining cyt*b*. Haplotype networks either grouped most samples together, or separated samples into two haplotypes largely correlated with geographic location. The corroboration of haplotype networks for ND2, which evolves at a relatively fast rate, and ATP8, a comparatively conserved gene region, strengthens the case for managing these subpopulations as two management units, as does the extremely low diversity found in the southern subpopulation. Further studies should include nuclear genes for comparison.

### Conservation implications

Synanthropic species, which cohabit with humans and benefit from resources and modifications that exist in anthropogenic landscapes, present unique challenges for conservation management. Today, at least 14 highly endangered birds are recognized as synanthropic, including the Bahama Oriole (Coulombe, Kesler & Gouni, 2011; Price, Lee & Hayes, 2011; Wright, Lake & Dolman, 2012). The extent to which synanthropy affects gene flow among populations remains unclear, but rapid cultural and evolutionary changes may be associated with landscape modification (Johnston, 2001; Boardman, 2006; Jiménez et al., 2013). The relatively recent clustering of Bahama Orioles around developed areas, where the favored breeding habitat occurs (introduced Coconut Palms), offers a useful model for future investigation of gene flow, particularly as habitat that may have been favored in the past for nesting has been devastated by human-caused forest fires (Price, Lee & Hayes, 2011).

Given the strong preference of Bahama Orioles for coconut palms in anthropogenic habitat during the breeding season, the arrangement of available nesting habitat may structure populations, even over small distances. This phenomenon has already been observed in natural systems, where ecological associations with forest or aquatic environments lead to population structuring over small distances (Arnaux et al., 2014; Valderrama et al., 2014). On Andros Island, human development is largely restricted to a narrow strip along the eastern coastline, and townships are separated by secondary pine forest and broadleaf coppice habitat. Thus, the small number of orioles known to nest on the west coast near a small fishing resort (Allen, 1890), which we were unable to sample, may have very limited gene flow with the other subpopulations due to geographic distance and unsuitable breeding habitat over much of the western portion of the island. Future studies examining genetic diversity in this taxon and changes over time should include samples from the west coast of Andros, as well as museum specimens not only from Andros, but also from Abaco Island, where the species formerly occurred.

For critically endangered species with restricted geographic ranges, stochastic natural disasters such as hurricanes pose a serious threat to species persistence (Fleischer et al., 2007). This threat is in addition to those already mentioned for the Bahama Oriole: lethal yellowing disease of the favored Coconut Palm nesting habitat, brood parasitism by the recently arrived Shiny Cowbird, and ongoing habitat loss (Price, Lee & Hayes, 2011). Translocation of individuals to other areas may decrease the probability of extinction by reducing vulnerability to natural disasters. Our study suggests that translocation of Bahama Orioles from Andros to Abaco Island, where the species formerly occurred, should include individuals from North Andros, Mangrove Cay, and South Andros to maximize genetic diversity in the translocated population. Translocated individuals may experience stress, delayed breeding, decreased clutch size, and increased mortality risk (Letty, Marchandeau & Aubineau, 2007; Dickens, Delehanty & Romero, 2010; Kaler et al., 2010). Inbreeding and diminished genetic diversity within the translocated population may also result (Jamieson, 2011). Thus, this conservation tool should be used with much caution and careful planning. Nevertheless, translocation can be an effective strategy to restore or supplement populations if individuals are carefully chosen, release areas are selected and prepared properly, and effective habitat management is practiced (Sutherland et al., 2010; Laws & Kessler, 2012).

## Additional Information and Declarations

### Field Study Permissions

The following information was supplied relating to field study approvals (i.e., approving body and any reference numbers): Bahamas Ministry of the Environment Permit to Conduct Scientific Research in the Bahamas (no ID number). Issued March 19, 2010 to William K. Hayes. USDA/APHIS/VS Permit 108969 to import tissue samples.

### DNA Deposition

The following information was supplied regarding the deposition of DNA sequences: GenBank accession numbers: ATPase 6/8: JN020618–JN020630; cytochrome *b*: JN020603–JN020617; NADH2: JN020589–JN020630.

## References

[ref-1] Allen JA (1890). Description of a new species of *Icterus* from Andros Island, Bahamas. Auk.

[ref-2] American Ornithologists’ Union (2010). Fifty-first supplement to the American Ornithologists’ Union Check-list of North American Birds. Auk.

[ref-3] Arctander P (1988). Comparative studies of avian DNA by restriction fragment length polymorphism analysis: convenient procedures based on samples from live birds. Journal of Ornithology.

[ref-4] Arnaux E, Eraud C, Navarro N, Tougard C, Thomas A, Cavallo F, Vetter N, Faivre B, Garnier S (2014). Morphology and genetics reveal an intriguing pattern of differentiation at a very small geographic scale in a bird species, the Forest Thrush *Turdus Iherminieri*. Heredity.

[ref-5] Baltz ME (1995). First records of Shiny Cowbird (*Molothrus bonariensis*) in the Bahama Archipelago. Auk.

[ref-6] Baltz ME (1996). The distribution and status of the Shiny Cowbird on Andros Island. Bahamas Journal of Science.

[ref-7] Baltz ME (1997). Status of the Black-cowled Oriole (*Icterus dominicensis northropi*) in the Bahamas. Report to the Department of Agriculture.

[ref-8] Boardman R (2006). The international politics of bird conservation: biodiversity, regionalism and global governance.

[ref-9] Cadena CD, Gutiérrez-Pinto N, Dávila N, Chesser RT (2011). No population structure in a widespread aquatic songbird from the Neotropics. Molecular Phylogenetics and Evolution.

[ref-10] Clement M, Posada D, Crandall KA (2000). TCS: a computer program to estimate gene genealogies. Molecular Ecology.

[ref-11] Cortes-Rodriguez N, Hernandez-Banos BE, Navarro-Seguenza AG, Omland KE (2008). Geographic variation and genetic structure in the streak-backed oriole: low mitochondrial DNA differentiation reveals recent divergence. Condor.

[ref-12] Cortéz-Rodríguez N, Jacobsen F, Hernandez-Baños BE, Navarro-Siguenza AG, Peters JL, Omland KE (2013). Coalescent analyses show isolation without migration in two closely related tropical orioles: the case of *Icterus graduacauda* and *Icterus chrysater*. Ecology and Evolution.

[ref-13] Coulombe GL, Kesler DC, Gouni A (2011). Agricultural coconut forest as habitat for the critically endangered Tuamotu Kingfisher (*Todiramphus gambieri gertrudae*). Auk.

[ref-14] Crandall KA, Templeton AR, Harvey PH, Leigh Brown AJ, Smith JM, Nee S (1996). Applications of intraspecific phylogenetics. New uses for new phylogenies.

[ref-15] Currie D, Wunderle JM, Ewert DN, Anderson MR, Davis A, Turner J (2005). Habitat distribution of birds wintering on Central Andros, The Bahamas: implications for management. Caribbean Journal of Science.

[ref-16] Dickens MJ, Delehanty DJ, Romero LM (2010). Stress: an inevitable component of animal translocation. Biological Conservation.

[ref-17] Excoffier L, Lischer HEL (2010). Arlequin suite version 3.5: a new series of programs to perform population genetics analyses under Linux and Windows. Molecular Ecology Resources.

[ref-18] Excoffier L, Smouse PE, Quattro JM (1992). Analysis of molecular variance inferred from metric distances among DNA haplotypes: application to human mitochondrial-DNA restriction data. Genetics.

[ref-19] Fetzner JW, Crandall KA (2003). Linear habitats and the nested clade analysis: an empirical evaluation of geographic versus river distances using an Ozark crayfish (Decapoda: Cambaridae). Evolution.

[ref-20] Fleischer RC, Slikas B, Beadell J, Atkins C, McIntosh CE, Conant S (2007). Genetic variability and taxonomic status of the Nihoa and Laysan Millerbirds. Condor.

[ref-21] Gaunt AS, Oring LW (1997). Guidelines to the use of wild birds in research.

[ref-22] Grant PR, Grant BR, Petren K (2001). A population founded by a single pair of individuals: establishment, expansion, and evolution. Genetica.

[ref-23] Hackett SJ (1996). Molecular phylogenetics and biogeography of Tanagers in the genus *Ramphocelus* (Aves). Molecular Phylogenetics and Evolution.

[ref-24] Hedrick PW (1998). Genetics of populations.

[ref-25] Jamieson IG (2011). Founder effects, inbreeding, and loss of genetic diversity in four avian reintroduction programs. Conservation Biology.

[ref-26] Jaramillo A, Burke P (1999). New World blackbirds: the Icterids.

[ref-27] Jiménez G, Meléndez L, Blanco G, Laiolo P (2013). Dampened behavioral responses mediate birds’ association with humans. Biological Conservation.

[ref-28] Johnston RF, Marzluff JM, Bowman R, Donnelly R (2001). Synanthropic birds of North America. Avian ecology and conservation in an urbanizing world.

[ref-29] Kaler RSA, Ebbert SE, Braun CE, Sandercock BK (2010). Demography of a reintroduced population of Evermann’s Rock Ptarmigan in the Aleutian Islands. Wilson Journal of Ornithology.

[ref-30] Larkin MA, Blackshields G, Brown NP, Chenna R, McGettigan PA, McWilliam H, Valentin F, Wallace IM, Wilm A, Lopez R, Thompson RD, Gibson TJ, Higgins DG (2007). Clustal W and Clustal X version 2.0. Bioinformatics.

[ref-31] Laws RJ, Kessler DC (2012). A Bayesian network approach for selecting translocation sites for endangered island birds. Biological Conservation.

[ref-32] Letty J, Marchandeau S, Aubineau J (2007). Problems encountered by individuals in animal translocations studies: lessons from field studies. Ecoscience.

[ref-33] Librado P, Rozas J (2009). DnaSP version 5: a software for comprehensive analysis of DNA polymorphism data. Bioinformatics.

[ref-34] Longmire JL, Lewis AK, Brown NC, Buckingham JM, Clark LM, Jones MD, Meincke LJ (1988). Isolation and characterization of a highly polymorphic centromeric tandem repeat in the family Falconidae. Genomics.

[ref-35] Lovette IJ, Bermingham E, Ricklefs RE (1999). Mitochondrial DNA phylogeography and the conservation of endangered Lesser Antillean *Icterus* orioles. Conservation Biology.

[ref-36] Milá B, Tavares ES, Saldaña AM, Karubian J, Smith TB, Baker AJ (2012). A trans-Amazonian screening of mtDNA reveals deep intraspecific divergence in forest birds and suggests a vast underestimation of species diversity. PLoS ONE.

[ref-37] Mitton JB (1998). Selection in natural populations.

[ref-38] Miyamoto MM, Fitch WM (1995). Testing phylogenies and phylogenetic methods with congruence. Systematic Biology.

[ref-39] Nei M (1987). Molecular evolutionary genetics.

[ref-40] Nixon KC, Carpenter JM (1996). On simultaneous analysis. Cladistics.

[ref-41] Omland KE, Lanyon SM, Fritz SJ (1999). A molecular phylogeny of the New World Orioles (*Icterus*): the importance of dense taxon sampling. Molecular Phylogenetics and Evolution.

[ref-42] Perdices A, Doadrio I (2001). The molecular systematics and biogeography of the European cobitids based on mitochondrial DNA sequences. Molecular Phylogenetics and Evolution.

[ref-43] Pook CE, Wuster W, Thorpe RS (2000). Historical biogeography of the Western Rattlesnake (Serpentes: Viperidae: *Crotalus viridis*), inferred from mitochondrial DNA sequence information. Molecular Phylogenetics and Evolution.

[ref-44] Price MR, Lee VA, Hayes WK (2011). Population status, habitat dependence, and reproductive ecology of Bahama Orioles: a critically endangered synanthropic species. Journal of Field Ornithology.

[ref-45] Robertson OJ, Radford JQ (2009). Gap-crossing decisions of forest birds in a fragmented landscape. Austral Ecology.

[ref-46] Saitoh T, Sugita N, Someya S, Iwami Y, Kobayashi S, Kamigaichi H, Higuchi A, Asai S, Yamamoto Y, Nishiumi I (2015). DNA barcoding reveals 24 distinct lineages as cryptic bird species candidates in and around the Japanese Archipelago. Molecular Ecology Resources.

[ref-47] Sgariglia EA, Burns KJ (2003). Phylogeography of the California Thrasher (*Toxostoma redivivum*) based on nested-clade analysis of mitochondrial-DNA variation. Auk.

[ref-48] Spielman D, Brook BW, Frankham R, Schaal BA (2004). Most species are not driven to extinction before genetic factors impact them. Proceedings of the National Academy of Sciences.

[ref-49] Sutherland WJ, Armstrong D, Butchart SH, Earnhardt JM, Ewen J, Jamieson I, Jones CG, Lee R, Newbery P, Nichols JD, Parker KA, Sarrazin F, Seddon PJ, Shah N, Tatayah V (2010). Standards for documenting and monitoring bird reintroduction projects. Conservation Letters.

[ref-50] Templeton AR, Crandall KA, Sing CF (1992). A cladistic analysis of phenotypic associations with haplotypes inferred from restriction endonuclease mapping and DNA sequence data. III. Cladogram estimation. Genetics.

[ref-51] Thurston G (2010). South Andros farm road progresses. http://www.bahamaslocal.com/newsitem/8010/South_Andros_farm_road_progresses.htm.

[ref-52] Valderrama E, Pérez-Emán JL, Brumfield RT, Cuervo AM, Cadena CD (2014). The influence of the complex topography and dynamic history of the montane Neotropics on the evolutionary differentiation of a cloud forest bird (*Premnoplex brunnescens*, Furnariidae). Biogeography.

[ref-53] White AW (1998). A birder’s guide to the Bahama Islands (including Turks and Caicos).

[ref-54] Wiens JJ (1998). Combining data sets with different phylogenetic histories. Systematic Biology.

[ref-55] Wiley JW (1985). Shiny cowbird parasitism in two avian communities in Puerto Rico. Condor.

[ref-56] Wright HL, Lake IR, Dolman PM (2012). Agriculture—a key element for conservation in the developing world. Conservation Letters.

[ref-57] Wunderle JM, Waide RB (1993). Distribution of overwintering nearctic migrants in The Bahamas and Greater Antilles. Condor.

